# Joint Analysis of Microbial and Immune Cell Abundance in Liver Cancer Tissue Using a Gene Expression Profile Deconvolution Algorithm Combined With Foreign Read Remapping

**DOI:** 10.3389/fimmu.2022.853213

**Published:** 2022-04-14

**Authors:** Dongmei Ai, Yonglian Xing, Qingchuan Zhang, Yishu Wang, Xiuqin Liu, Gang Liu, Li C. Xia

**Affiliations:** ^1^Basic Experimental Center of Natural Science, University of Science and Technology Beijing, Beijing, China; ^2^School of Mathematics and Physics, University of Science and Technology Beijing, Beijing, China; ^3^National Engineering Laboratory for Agri-Product Quality Traceability, Beijing Technology and Business University, Beijing, China; ^4^School of Mathematics, South China University of Technology, Guangzhou, China

**Keywords:** tumor microenvironment, RNA-seq, gene expression profiling, support vector regression, particle swarm algorithm

## Abstract

Recent transcriptomics and metagenomics studies showed that tissue-infiltrating immune cells and bacteria interact with cancer cells to shape oncogenesis. This interaction and its effects remain to be elucidated. However, it is technically difficult to co-quantify immune cells and bacteria in their respective microenvironments. To address this challenge, we herein report the development of a complete a bioinformatics pipeline, which accurately estimates the number of infiltrating immune cells using a novel Particle Swarming Optimized Support Vector Regression (PSO-SVR) algorithm, and the number of infiltrating bacterial using foreign read remapping and the GRAMMy algorithm. It also performs systematic differential abundance analyses between tumor-normal pairs. We applied the pipeline to a collection of paired liver cancer tumor and normal samples, and we identified bacteria and immune cell species that were significantly different between tissues in terms of health status. Our analysis showed that this dual model of microbial and immune cell abundance had a better differentiation (84%) between healthy and diseased tissue. *Caldatribacterium* sp., *Acidaminococcaceae* sp., *Planctopirus* sp., *Desulfobulbaceae* sp.,*Nocardia farcinica* as well as regulatory T cells (Tregs), resting mast cells, monocytes, M2 macrophases, neutrophils were identified as significantly different (Mann Whitney Test, FDR< 0.05). Our open-source software is freely available from GitHub at https://github.com/gutmicrobes/PSO-SVR.git.

## Introduction

Contrary to the intuition of most people, bacteria are present in almost every part of the human body, with over 1,000 species in the gut alone. Our commensal bacteria maintain a dynamic balance with each other, and their imbalance can lead to a variety of diseases in the human body, including many cancers (1). Indeed, approximately 20% of all lethal cancers in humans are induced by or associated with microorganisms (2). As a component of the tumor microenvironment (TME), bacteria can actively promote tumor development, as well as autoimmunity, contributing to mortality (3, 4).

Liver cancer is a malignant tumor and approximately 780,000 people were diagnosed with liver cancer worldwide yearly (5). Previous studies analyzed microbes inferred from the whole-genome sequencing data of liver cancer biopsies. They revealed a strong association between the occurrence of liver cancer and certain bacterial flora. For examples: Moffatt et al. found that the bacterium *Porphyromonas gingivalis* promotes hepatocellular carcinoma by affecting host cell signaling and thus cytokine response, cell cycle, and apoptosis (6); Garner et al. found that methoxysterigmatocystin, O-methylsterigmatocystin, and other metabolites induced DNA repair-deficient bacterial lesions and thus initiates hepatocellular carcinogenesis (7); Mangul et al. found that *Escherichia coli*, *Streptococcus faecalis*, and *Clostridium parvum* could act together to significantly promote liver tumorigenesis. However, such activity could be inhibited by the addition of intestinal bacteria (e.g., *Bifidobacterium longum* and *Lactobacillus acidophilus*) and rectal fungi (8).

However, these studies were mostly focused on the effects of intestinal bacteria upon hepatocellular carcinoma (HCC) while few have examined the bacteria present within hepatocellular carcinoma tissues. Tumor-infiltrating bacteria could be analyzed using sequencing reads of non-human source, however, these unmapped foreign reads are often overlooked. In fact, it is possible to accurately estimate microbial abundance in tissue biopsies using foreign reads remapping (9). In this study, we applied our previously developed GRAMMy (10) tool to identify the bacteria that are associated with liver cancer by adding a series of analyses on unmapped foreign reads filtered from RNA-seq data so as to estimate the relative abundance of infiltrating bacteria present within the tissue.

Studies have also shown that the infiltration of various immune cell populations, including monocytes/macrophages, natural killer cells (NK), NKT cells and T cells, is the main pathogenic feature for oncogenesis or other lesions in liver (11). Rohr-Udilova et al. observed considerable differences in the composition of immune cells between HCC and healthy liver. Pushpa Hegde et al. found a decrease in circulating mucosal-associated invariant T cells in patients with alcoholic or nonalcoholic fatty liver disease-related cirrhosis (12). Functional immune level changes were detected in a group of healthy people and liver transplant recipients (13). Microbial infection is very likely to occur in the clinical treatment of liver diseases, especially in the treatment of liver transplantation; bacterial infection being the most common (incidence of 31.45%) (14). Monitoring the immune status of transplant recipients is essential to predict the risk of infection.

Thus, researchers have just begun to understand the regulatory role of bacteria in the development of cancer, as well as oncogenesis at the interface of bacteria/immune cell interaction. Immune cells alone are a key component of the TME and play a critical role in cancer development and immunization. Therefore, in order to understand the dynamics of the TME, it is equally important to understand the presumed synergistic dynamics between bacteria and tissue-infiltrating immune cells. Computational deconvolution methods have become a convenient choice to assess tissue-infiltrating immune cells by formulating the problem as a system of equations used to describe the gene expression of a sample as a weighted sum of the expression profiles of mixed cell types. That is, once given the immune cell-type characteristic matrix and overall gene expression, by solving the deconvolution problem, immune cell types and levels can be reasonably co-quantified with bacteria using mRNA-seq data without resorting to additional experiments.

The deconvolution problem could be solved in several ways, such as by Support Vector Regression (SVR) (e.g., CIBERSORT) (15), linear least squares regression (e.g., TIMER) (16) and constrained least squares regression (e.g., EPIC and MCP-counter) (17, 18). CIBERSORT uses v-SVR linear regression to solve the linear equation model based on microarray data (RNA-seq data can also be used). The coefficient of the regression model represents the relative proportion of 22 immune cell types. TIMER calculates the abundance of six immune cells including CD4 T cells, CD8 T cells and B cells based on the constrained least square method, which better solved the multicollinearity problem caused by high-dimensional features. EPIC uses least square regression to infer the mRNA number of six immune cells and other cell types, and then converts the mRNA number into the relative proportion of related immune cells. Finally, through the verification of tumor active genes and clinical data of many patients, it is found that epic results are clinically applicable. Finally, the murine Microenvironment Cell Population counter (MCP-counter) was based on highly specific transcriptomic markers, allows a robust quantification of the count number of eight immune cell types and two stromal cell populations in heterogeneous tissues based on transcriptomic data, which represent the cell content.

At present, the main method of optimizing the SVR deconvolution problem uses an heuristic algorithm. Particle swarm optimization (PSO) is simple and easy to operate algorithm, and the optimization search process of PSO take into account the local search ability and global search ability, which can greatly improve the accuracy of SVR solution. At present, PSO- SVR algorithm has been applied in many fields. For example, Mingcong Deng et al. applied PSO- SVR algorithm to robotics in 2014 to predict the ball receiving time of a robot player (19). In 2017, the same team used PSO to optimize the parameters of the generalized Gaussian kernel model and confirmed that the algorithm also shows good performance in a pneumatic bending rubber actuator control system (20). However, few teams have applied PSO- SVR to the field of tumor immune cell infiltration. This study aims to further study the universality of this algorithm and its advantages or limitations compared with other algorithms in this field through the results of liver cancer samples under this algorithm.

Mohammadi et al. evaluated several methods for solving the deconvolution problem and found that combining the loss function with regularization can improve the solution performance in the presence of highly correlated cell types in the mixture (21). However, the effect of the size of the regularization parameters on the effect of immune cell counting is unknown and the SVR model accuracy is affected by the initial parameters, such as the kernel function coefficients and penalty factors. To increase the robustness of the algorithm and reduce the influence of the initial self-defined parameters on the proportional counting results of immune cells, we introduced the particle swarm optimized support vector regression (PSO-SVR) algorithm, which uses PSO -a powerful parameter iterative optimization solution algorithm to improve the accuracy of the SVR solution. The algorithm was also used to compare with CIBERSORTX (22), EPIC, and MCP-counter on three real data sets, which confirmed its better accuracy.

Finally, we used the PSO-SVR algorithm and foreign read remapping to analyze a collection of paired liver cancer tumor and normal samples, identified bacteria and immune cell species that are significantly different between samples, and evaluated their joint effects by predicting the tumoral or normal pathological status of their originating tissue. The joint model identified B cells, T cells and *Caldatribacterium* sp., *Magnetobacteriaceae* sp. as pathological markers and together they were powerfully predictive for the pathologic status. Based on results from the three cases, classification accuracy when using only a single input feature is lower - only bacteria: 0.70, only immune cell feature: 0.74, and both features: 0.84. Such preliminary results are useful in resolving standing low response issue of immune checkpoint inhibitor-based immunotherapy (23, 24), in that microbial markers are potentially actionable targets (25, 26). As the joint immunomodulatory effects of the microbiome and immune cells are further elucidated for other cancer types, we could expect to find more novel biomarkers that could be intervened upon to improve normal tissue’s immune response against tumor.

## Materials and Methods

### The Liver Cancer mRNA Sequence Data Set

**Figure 1:**
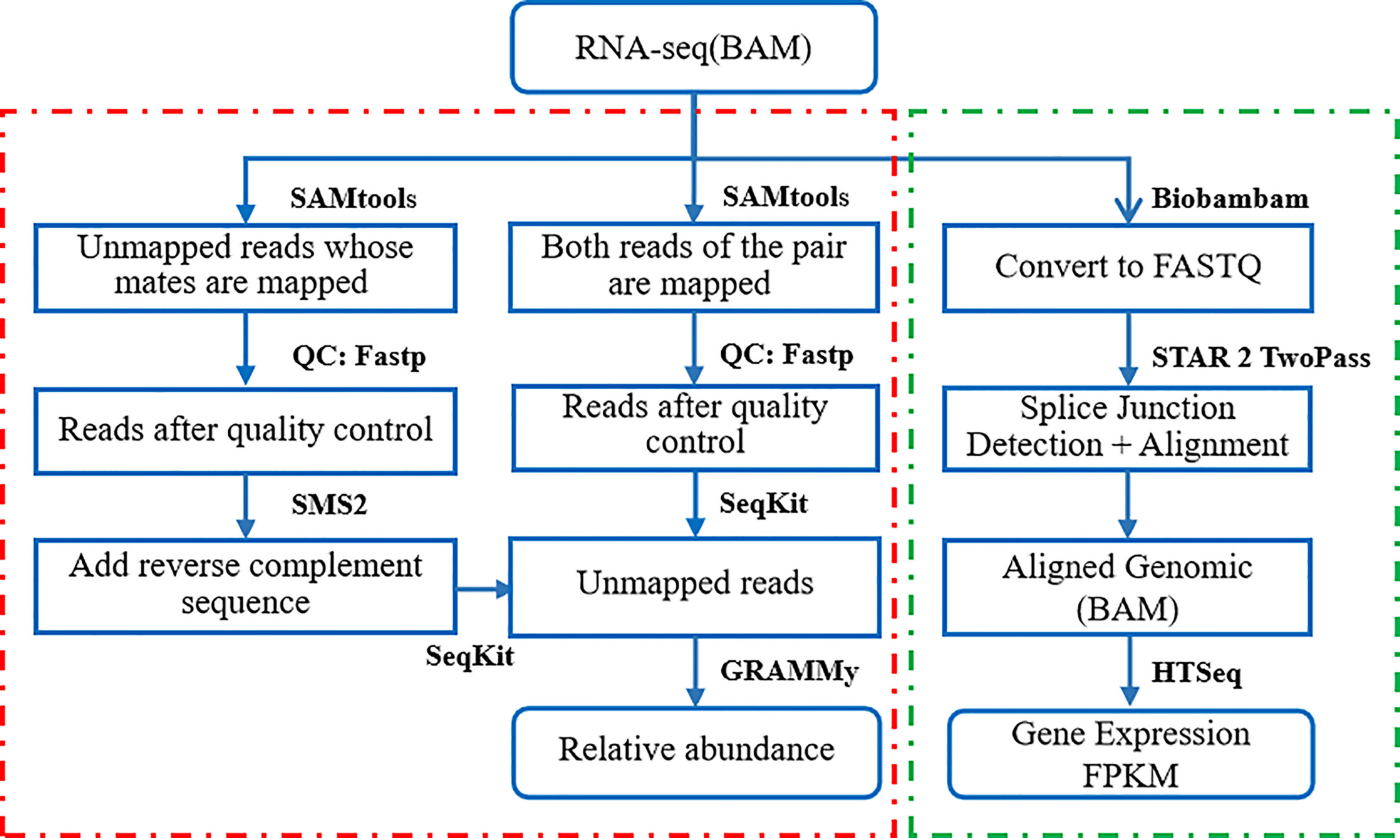
Flow chart of RNA-Seq sequencing data processing.

The mRNA-seq raw sequence data in BAM format of liver cancer patients’ normal and tumor tissue biopsies for this study were downloaded from the Seven Bridges Cancer Genomics Cloud (CGC https://www.cancergenomicscloud.org/). The data set included 98 samples (49 pairs of RNA-Seq sequencing samples of primary liver cancer tumor tissue and adjacent normal tissue). The read sequences that did not map to the reference genome GRCH38 were extracted from the BAM files. These foreign reads were then mapped to the RefSeq (NCBI Reference Sequence Database) (https://www.ncbi.nlm.nih.gov/refseq/) - a large collection of bacterial genomes. The mapping results were then input to the GRAMMy pipeline for the relative abundance estimation of infiltrating bacteria. The obtained expression and microbial profiles were used for downstream analysis. The data processing flow is shown in **Figure 1**.

We first filtered the reads that were not matched to the human genome reference GRCH38 from the RNA-Seq samples in BAM format with SAMtools, which included reads that were not mapped at both ends and reads that were not mapped at one end (mapped at one end but not at the other). Apply Fastp to remove low-quality reads and partial reads, and excise poor quality bases. Use the BWA tool to remap the unmapped reads to a comprehensive microbial reference library RefSeq, containing the full genome sequence of 23,790 bacteria and archaea. We then applied GRAMMy algorithm to estimate the bacteria relative abundance.

The mRNA analysis pipeline begins with the Alignment Workflow, which is performed using a two-pass method with STAR. STAR aligns each read group separately and then merges the resulting alignments into one. Following alignment, BAM files are processed through the RNA Expression Workflow to determine RNA expression levels. The reads mapped to each gene are enumerated using HT-Seq-Count. Expression values are provided in a tab-delimited format.

### Validation Data Sets for ImmuneCell Infiltration

Three real data sets were used to validate the PSO-SVR algorithm as compared to the CIBERSORTX, EPIC, and MCP-counter algorithms. The first validation data set from the CIBERSORT article consists of the gene expression profiles of 20 peripheral blood mono nuclear cells (PBMC) samples and the ratio of immune cells determined through flow cytometry for the same samples. The second data set comes from the National Center for Biotechnology Information Search database (NCBI database) under the GEO Datasets GSE64385. It consists of the gene expression profile of 10 colorectal cancer (CRC) samples and the composition of immune cells as determined by immunohistochemistry of the same samples. The third data set from CIBERSORTX consists of the gene expression profiles of 19 melanoma samples and the composition of immune cells obtained through single-cell sequencing technique of the same samples. The full description of these data sets is shown in **Supplementary Table 1**.

### Extracting Unmapped Reads

We first filtered the reads that did not matched to the human genome reference GRCH38 from the RNA-Seq samples in BAM format using SAMtools (27). The filtered reads included those that were not mapped at both ends and those that were not mapped at one end (mapped at one end but not the other). The process was as follows:

Extracting unmapped single reads: we used the command “samtools view -u -f 4 -F 264” to extract single-end unmapped reads in the format of FASTQ (28).

Extracting unmapped paired-end reads: we used the command “samtools view -u -f 12 -F 256” to extract paired-end unmapped reads with the format FASTQ.

### Sequencing Quality Control

Sequencing quality issues like low-confidence bases and sequence-specific bias complicate mRNA-seq analyses (29). We applied comprehensive quality control (QC) before analyses (30). We applied Fastp (31) to remove low-quality reads and partial reads, and to excise poor quality bases.

For the extracted unmapped paired-end reads, we used the command “fastp -q 0 -u 100 -n 10 -l 36 -A -G -M 0 -i” to delete sequences with base quality lower than 40% of Q15, sequences with N greater than 5, sequences with length less than 36, and broke up sequences, respectively. For the extracted unmapped single reads, we used the command “fastp -q 0 -u 100 -n 10 -l 36 -A -G -M 0 -i” to perform the same QC process on single-ended sequenced sequences. To merge the extracted single-end and paired-end unmapped reads, we used the SMS2 (32) software to add a reverse complementary sequence to the unmapped single reads. We then converted the resulting FASTQ formatted reads into the FASTA (33) format through SeqKit (34).

We then used the BWA tool (35) to remap the unmapped reads to a comprehensive microbial reference library RefSeq, containing the full genome sequence of 23,790 bacteria and archaea. We then applied the GRAMMy algorithm to estimate the relative abundance of bacteria to mitigate the problem of ambiguous mapping of short reads to relative reference sequences.

### Statistical Analysis

The Mann Whitney U test, also known as “Mann Whitney rank sum test”, was proposed by H.B.mann and D.Rwhitney in 1947. It assumes that any two samples are from two populations that are exactly the same except the population mean, in order to test for significant difference between the mean of the two populations.

Assuming that the mean values of two populations exist, they are recorded as *μ*_1_,*μ*_2_ respectively. With only one translation difference between *f*_1_ and *f*_2_ at most, we get: *μ*_1_ = *μ*_2_ – α. The assumptions to be tested are as follows:


$$\{\begin{array}{l} H_{0}:\mu_{1}=\mu_{2},H_{1}:\mu_{1}<\mu_{2}\\ H_{0}:\mu_{1}=\mu_{2},H_{1}:\mu_{1}>\mu_{2} \end{array}$$

The steps of Mann Whitney U test are:

1. Randomly select two independent random samples with capacity of *N_A_* and *N_B_* from two populations A and B, arrange (*N_A_* + *N_B_*) observations in order of size. If the same observations exist, the average of their bit order is used.
2. Calculate the grade and *T_A_* and *T_B_* of two samples.
3. The formula of Mann Whitney U test can be given according to *T_A_* and *T_B_*. The two calculated U values are not equal, but their sum is always equal to *N_A_N_B_*, that is, *U_A_* + *U_B_* = *N_A_N_B_*. If *N_A_* < 20 and *N_B_* < 20, the test statistics are:

$$U_{A}=N_{A}N_{B}+N_{A}(N_{A}+1)/2-T_{A}$$

$$U_{B}=N_{A}N_{B}+N_{B}(N_{B}+1)/2-T_{B}$$

In the test, because the critical value table of Mann Whitney U test only gives a smaller critical value, the smaller U value in *U_A_* and *U_B_* is used as the test statistic.

4. Select the smaller U value to compare with the critical value of U. if *U* > *U_α_* (*α* = 0.05, accept the original assumption *H*_0_. If *U* < *U_α_* (*α* = 0.05, Then reject *H*_0_ and accept *H*_1_. The acceptance domain is the same as Wilcoxon test. U test can also be divided into small samples and large samples. In case of small samples, the critical values of U have been compiled into a table. In large samples, the distribution of U tends to be normal, so it can be treated by normal approximation.

The loss function is an index to measure the performance of the prediction model in predicting the expected results. The commonly used loss functions are mean squared error (MSE) and root mean squared error (RMSE). Since MSE squares the error (y – y^predicted = e), if e > 1, the value of the error will increase a lot. If there is an outlier in our data, the value of e will be very high and will be much greater than │e│. This will make the model with MSE loss give a higher weight to outliers. In order to minimize this outlier data point, we use the RMSE value, namely root mean square error, but at the expense of the prediction effect of other normal data points, which will eventually reduce the overall performance of the model.


$$RMSE={\left(\sum_{i=1}^{n}\frac{{\left(\hat{y}-y_{i}\right)}^{2}}{n}\right)}^{\frac{1}{2}}$$

Correlation analysis refers to the analysis of two or more variable elements with correlation, so as to measure the correlation degree of two variable factors Correlation analysis can be carried out only when there is a certain connection or probability between the elements of correlation.

(1) Pearson correlation coefficient

Given two continuous variables x and y, the Pearson correlation coefficient is defined as:


$$\rho =\frac{\Sigma_{i=1}^{N}(x_{i}-\bar{x})(y_{i}-\bar{y})}{{\left[\Sigma_{i=1}^{N}{\left(x_{i}-\bar{x}\right)}^{2}\Sigma_{I=1}^{N}{\left(y_{i}-\bar{y}\right)}^{2}\right]}^{\frac{1}{2}}}$$

Where $\bar{x}$ and $\bar{y}$ are the mean values of the variables x and y respectively.

ρ close to 0 indicates that there is no correlation between the two variables; whereas close 1 or - 1 indicates that the two variables are strongly correlated.

(2) Spearman correlation coefficient

Spearman correlation coefficient is defined as Pearson correlation coefficient ρ between hierarchical variables. For samples with a sample size of N, N original data are converted into hierarchical data. Compared with Pearson correlation coefficient, Spearman correlation coefficient is insensitive to data errors and extreme values, which is defined as.


$$\rho_{s}=\frac{\Sigma_{i=1}^{N}(R_{i}-\bar{R})(S_{i}-\bar{S})}{{\left[\Sigma_{i=1}^{N}{\left(R_{i}-\bar{R}\right)}^{2}\Sigma_{I=1}^{N}{\left(S_{i}-\bar{S}\right)}^{2}\right]}^{\frac{1}{2}}}$$

Where R and S are the grades of observed values i respectively, $\bar{R}$ and $\bar{S}$ are the average grades of variables x and y respectively, and N is the total number of observed values.

Logistic regression, also known as log probability regression, is a machine learning method used to solve the binary classification problem, which is used to estimate the possibility of something. It does not need to scale the input features, and the interpretability of the model is very good. The influence of different features on the final result can be seen from the weight of features. We fit the following regularization model to binary features as:


$$\min_{\beta_{0},\beta }-\left[\frac{1}{n}\sum_{i=1}^{n}y_{i}(\beta_{0}+x_{j}^{T}\beta )-\log(1+e^{\beta_{0}+x_{j}^{T}\beta })\right]+\mu \left[\frac{(1-\alpha ){\left||\beta |\right|}_{2}^{2}}{2}+\alpha {\left||\beta |\right|}_{1}\right]$$

Where β is the regression coefficient. Parameter α is the balanced lasso (L1) and ridge (L2) regularization, and λ determines their weights.

Therefore, we use the logistic regression classifier to classify 98 samples. Their corresponding bacterial relative abundance data and immune cell proportion data were used as input characteristics, and the sample status (normal/tumor) was used as the prediction variable 0 or 1. 75% of the data were used for training and 25% for testing. The association between hepatocarcinogenesis and tumor bacteria and invasive immune cells was analyzed through the classification results under different input characteristics.

## Results and Discussion

### The Particle Swarm Optimized - Support Vector Regression Algorithm

**Figure 2:**
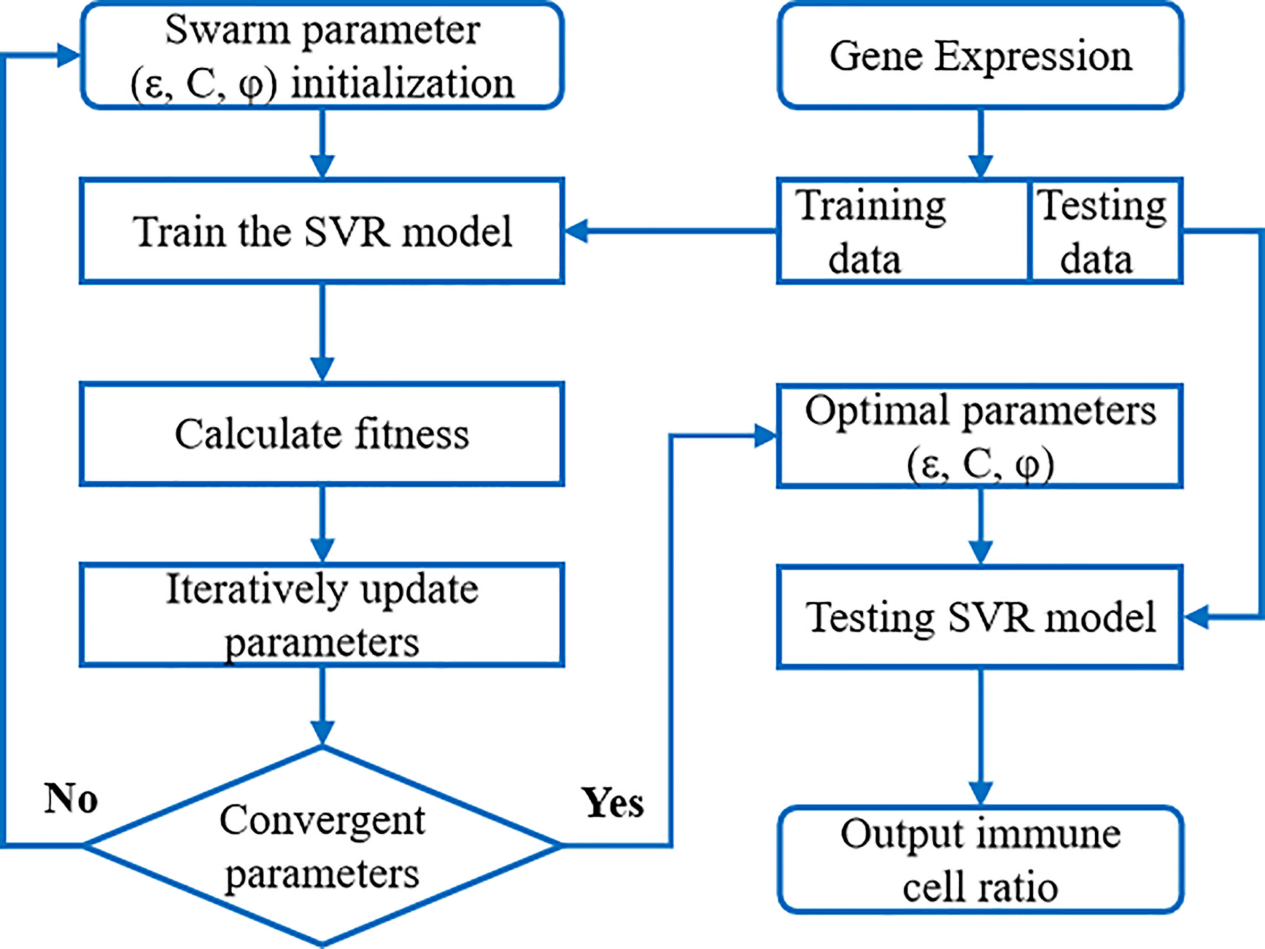
The flow chart of PSO-SVR algorithm. SVR model was embedded into the PSO algorithm to calculate the optimal parameters; then SVR model with optimized parameters was applied to the immune cell ratios.

Support Vector Regression (SVR) is a commonly used technique for non-linear unsupervised learning. The generalization ability and prediction accuracy of SVR depend on the choice of kernel function coefficients and penalty factors. Due to the large feature size, small sample size and unknown sample distribution, in order to avoid over fitting as much as possible, we used a simple and effective linear kernel function in order to avoid overfitting as much as possible. We introduced the particle swarm optimization (PSO) technique, which uses a powerful algorithm to invoke an iterative approach to solve parameter optimization. It can improve the accuracy of SVR results, and increase the robustness of the results through multiple iterations. The overall flowchart is shown in **Figure 2**.

We briefly introduce the SVR model for immune cell infiltration estimation. Based on a large amount of tissue gene expression profile data combined with *a priori* knowledge of purified leukocyte subpopulation expression profile data (i.e., a “signature matrix” representing the expression profile data of each gene in different immune cells), the proportion of immune cells in tumor biopsies can be accurately estimated. The idea was first used in CIBERSORT. The gene expression profile data were first transformed into a linear combination of marker genes for immune cells to solve for f (representing the proportion of immune cells) in a linear combination equation. The linear combination equation was expressed as:


$${\text{X}}_{\text{n}\times \text{L}}={\text{S}}_{\text{n}\times \text{m}}\times {\text{f}}_{\text{m}\times \text{L}}$$

Where X_n×L_ denotes the expression profile data of n genes from L samples for deconvolution. f_m×L_ is the proportion of immune cells to be sought, specifically indicating the proportion of each immune cell in L samples among the m immune cell types. S_n×m_ is the signature matrix, indicating the expression profile data of n genes in each immune cell for m immune cells. The SVR algorithm solves the linear combinatorial equation using a deconvolution scheme.

The accuracy of the immune cell ratio calculated through the above SVR model is influenced by the model parameters such as the penalty factor C, sensitivity ϵ, and kernel function coefficient ϕ. These parameters are crucial to an SVR model’s accuracy and generalizability. However, in practice, these parameters are largely manually chosen without justification. Here we propose to optimize (ϵ, C, ϕ) parameters through iterations using the particle swarm algorithm. In each iteration, the swarming particles update their velocity and position vectors by tracking two “optimal values” (p_ibest_, g_best_), where p_ibest_ denotes the individual optimal value of the particle and g_best_ is the global optimal value. In the computation, the expression data of each gene (i.e., each row vector in the *X_n_*_×_*_L_* matrix) was treated as a particle in the swarming iteration.


$${\text{v}}_{\text{i}+1}={\text{qv}}_{\text{i}}+{\text{c}}_{1}{\text{r}}_{1}({\text{p}}_{\text{ibest}}-x_{\text{i}})+{\text{c}}_{2}{\text{r}}_{2}({\text{g}}_{\text{best}}-x_{\text{i}})$$

$${\text{x}}_{\text{i}+1}=x_{\text{i}}+{\text{v}}_{\text{i}+1}$$

Where v_i_ and x_i_ represent the velocity vector and position vector of the i^th^ particle respectively, each gene is regarded as a particle respectively, and n represents the size of the population, specifically the number of genes; q is a non-negative inertia factor. The larger the value of q is, the stronger the global optimization ability is and the weaker the local optimization ability is; c_1_ and c_2_ are learning factors, general c_1_ = c_1_ = **2**; r_1_ and r_1_ both represent random coefficients belonging to [0,1]; p_ibest_ represents the individual optimal value of the ith particle, and g_best_ is the global optimal value. We provide more detailed parameter explanation and algorithm flow in the **Supplementary Materials**. **Table 1** provides an explanation of key parameters.

**Table 1:** PSO-SVR model parameter.

Parameter	Meaning	Reference range	Value
k(x,y)	kernel function	linear	k (x,y)=x·y
C	Penalty factor	[1,108]	3
ϵ	sensitivity	[0,0.2]	1.203564×10^-5^
Φ	Kernel function coefficient	[0.01,2.0]	0.04545455

We apply this iterative optimization process of particle swarm to solve the SVR model, so that its optimal input parameters are optimized according to the swarm fitness metric. Upon initialization, we specify the approximate ranges of the parameters ϵ, C, and ϕ as: ϵ = (0, 0.2), C = (1, 100), ϕ = (0.01, 2.0), and q_max_ and q_min_ values are 0.9 and 0.4 for the model. Upon convergence, we find the optimal parameter solution (ϵ, C, ϕ). SVR models using optimal parameters are validated using benchmark data sets. We apply model with these parameters to the expression data of liver cancer patients’ tissues to solve for immune cell infiltration ratios.

The major innovation of PSO-SVR over previous SVR deconvolution methods is that it applies the machine learning technique – particle swarm algorithm, to do SVR iterative optimization. In the process, the swarming algorithm searches for SVR hyperplane formed by support vectors that capture as many data points as possible while satisfying the given constraints and avoiding overfitting, using a linear “ω-insensitive” loss function. The function penalizes only those data points outside a specific error radius. The support vectors were selected from the signature matrix of genes, and the standard reference expression profile (signature matrix) was selected from a composition of 22 immune cells including CD4 T, and CD8 T immune cells in CIBERSORTX, but other signature gene sets can be applied as well.

### Benchmark of the PSO-SVR Algorithm

We gathered three data sets (see **Supplementary Table 1**), in which the immune cell proportions were determined through orthogonal flow cytometry (PBMC-FC), immunohistochemistry (CRC-IC), and single-cell RNA sequencing technologies (Melanoma-scRNA). Flow cytometry was an established technology to identify and determine different cell types in heterogeneous cell populations (36). immunohistochemistry is also well-established, using chemical reaction to label antibodies and to identify and quantify antigens within tissue cells. The genetic heterogeneity of cells of the same tissue can be also analyzed by scRNA-Seq at the level of individual cells to cluster and compute the composition immune cells (37). In order to verify the accuracy of PSO-SVR algorithm, we used data sets based on these technologies as the orthogonal control, and studied the deviation between PSO-SVR results and the control.

**Figure 3:**
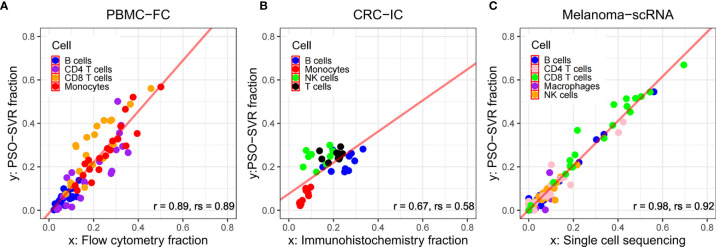
Comparison between PSO-SVR and orthogonal technologies. Scatter plots of immune cell fractions with regression of **(A)** PBMC-FC; **(B)** CRC-IC; and **(C)** Melanoma-scRNA data; r for Pearson’s correlation, rs for Spearman’s correlation.

As shown in **Figure 3A**, for the PBMC-FC data, that the PSO-SVR estimated B cell, CD4 T cells, CD8 T cells and Monocytes levels were very close to the flow cytometry results. Overall, the four types of immune cells present a good correlation. The CD8 T cells showed relatively less consistency as compared to the other two types, maybe because CD8 T cells accounted for a relatively small number of T lymphocytes in PBMC (5%–20%) thus is subject to less accurate estimation. As shown in **Figure 3B**, the PSO-SVR estimates of B cells, monocytes, NK cells, and T cells were also in good concordance with the immunohistochemistry results. As shown in **Figure 3C**, PSO-SVR estimated immune cell fractions, such as CD4 T and CD8 T cells, also showed a good correlation with the scRNA-seq levels.

To further evaluate the PSO-SVR estimates using the PBMC-FC, CRC-IC and Melanoma-scRNA data sets, we used three metrics, namely root mean square error (RMSE), Pearson correlation coefficient, and Spearman’s correlation coefficient. The individual indicators are shown in **Table 2**.

**Table 2:** Immune cell error table of three data sets.

	Cell type	Root mean square error (RMSE)	Pearson (r)	Spearman (rs)
**PBMC-FC**	B cells	0.028763	0.69534	0.67669
	Monocytes	0.056215	0.92192	0.93684
	CD4 T cells	0.073478	0.84827	0.88270
	CD8T cells	0.110915	0.84207	0.82105
**CRC-IC**	B cells	0.057609	0.39181	0.51515
	Monocytes	0.018876	0.87587	0.78181
	NK cells	0.116719	0.23098	0.30909
	T cells	0.060646	0.18626	0.16363
**Melanoma-scRNA**	B cells	0.019590	0.99128	0.85381
	Macrophages	0.028086	0.70316	0.67192
	NK cells	0.021296	0.92560	0.86315
	CD4 T cells	0.034945	0.95508	0.92847
	CD8 T cells	0.050518	0.97476	0.97543

We observe in **Table 2** that the error was generally larger for B cells and that both Pearson and Spearman’s correlation coefficients were relatively low. For CD4 T and CD8 T cells, the difference between the results presented in the PBMC-FC and Melanoma-scRNA is very small, and the RMSE and correlation coefficients for both are close, proving the reliability of the algorithm in this study.

Finally, the results calculated through PSO-SVR were compared with other algorithms such as CIBERSORTX, EPIC, MCP-counter, for calculating the proportion of immune cells in infiltrating tumors. From the results of PBMC-FC and Melanoma-scRNA (refer to **Supplementary Tables 2** and **5**), it can be observed that PSO-SVR outperforms CIBERSORTX, EPIC, MCP-counter, in terms of the three indicators, RMSE, Pearson, and Spearman correlation coefficients. However, the results of CRC-IC (refer to **Supplementary Table 3**) are not as good as the other three methods, which could be attributed to the small amount of CRC-IC data. Overall, our algorithm was highly accurate.

### Joint Microbial- and Immune-Effect Analysis of Hepatocellular Carcinoma Samples

#### Differential Presence of Infiltrating Immune Cells Between Tumor and Normal Tissues

We analyzed the differences in the relative proportions of immune cells in tumor samples and normal solid tissue samples. The non-parametric Mann-Whitney-Wilcoxon test was conducted in R software and then corrected for p-values with the Benjamini-Hochberg correction (FDR). Immune cells with significant differences were identified as shown in **Supplementary Table 5** (FDR < 0.05).

**Figure 4:**
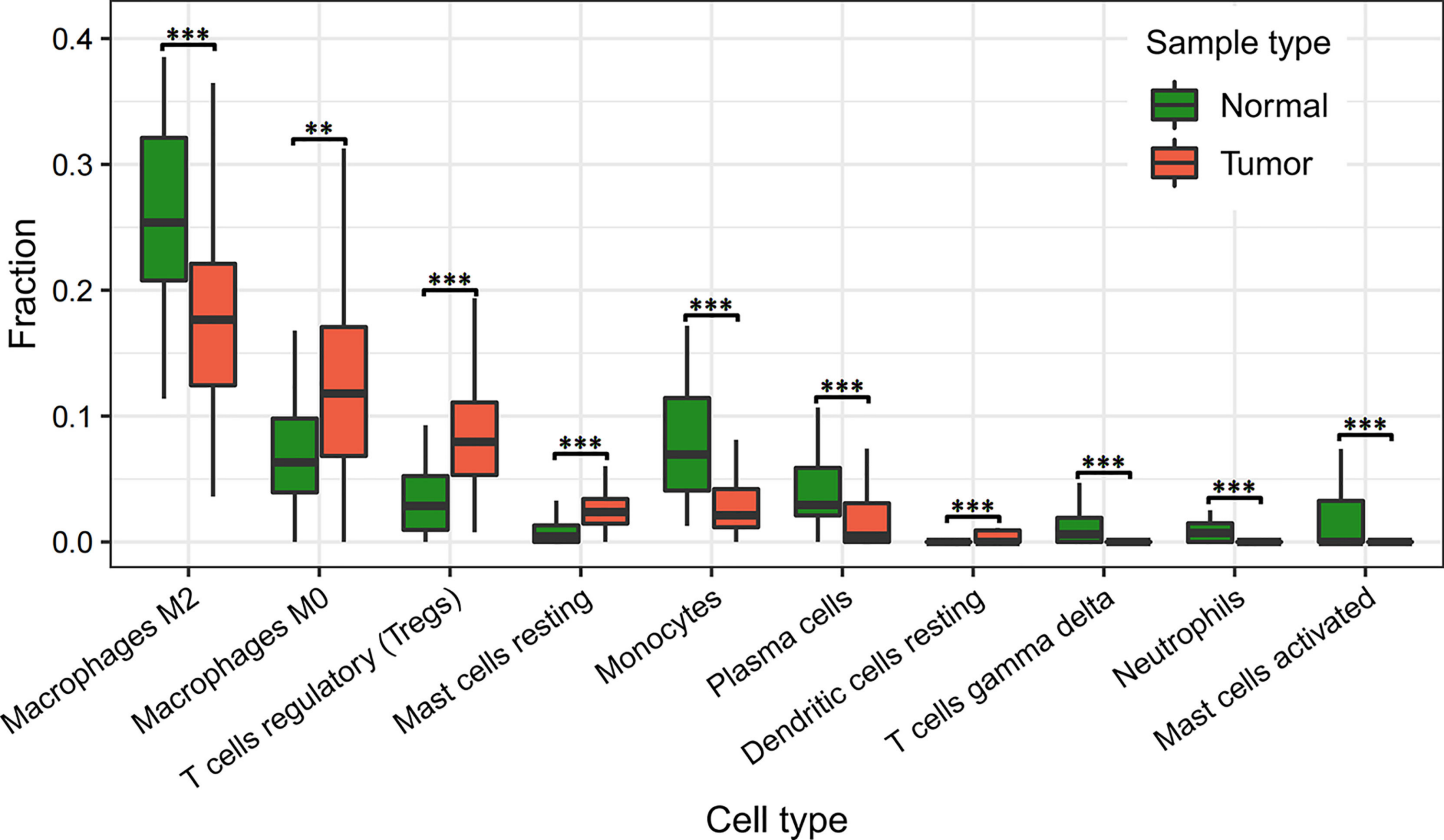
Differetial analysis of immune cell infiltration in normal and tumor tissues. “**” indicates FDR < 0.01; “***” indicates FDR < 0.001.

As it can be seen in **Figure 4** and **Supplementary Table 6**, the relative proportions of regulatory T cells [confidence level=95%, FDR= 1.58E-07] and 59.87% significantly higher in tumor tissues, and the relative proportions of Monocytes and Neutrophils are 150.07% and 363.69% significantly lower [FDR= 6.77E-07, 1.37E-05], as compared to normal solid tissue samples. These results suggested that regulatory T cells, Monocytes and Neutrophils were attracted toward tumor tissues who may act as host defense against invasive tumor growth. These findings were consistent with external knowledge and evidence.

As known, neutrophils provide the first line of defense for the innate immune system by phagocytosing, killing, and digesting bacteria and fungi (38). CD8 T cells are an important component of the immune system, and inducing an effective memory T cell response is a major target for vaccines against chronic infections and tumors (39). Studies have shown that the presence of Tregs in tumors of patients with hepatocellular carcinoma are suppressor cells and their increased levels are associated with immunosuppression and evasion in patients with cancer, where the inappropriate immune responses can be prevented by suppressing immune effector cells. In addition, the frequency of Tregs in lymphoid tissue, peripheral blood, and in the TME is greater than that in normal tissue (40).

#### Differential Presence of Infiltrating Bacteria Between Tumor and Normal Tissues

**Figure 5:**
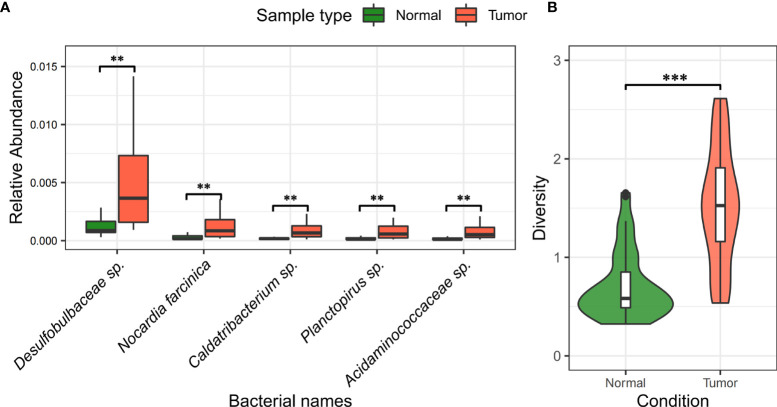
Difference and diversity of bacteria in liver cancer samples. **(A)** Differentiation of bacteria between liver tumor tissue and normal tissue. “**” indicates FDR < 0.05; “***” indicates FDR < 0.001. **(B)** Analysis of alpha diversity of bacteria in tumor and normal tissues. The green color on the left indicates normal samples, the red part on the right indicates tumor samples, and the middle BASE part indicates the interquartile range.

In **Table 3** and **Figure 5A**, we showed the most significant differentially present bacteria between tumor and normal tissues. These include *Caldatribacterium* sp. [↑74.94%, FDR=3.15×10^-11^], *Acidaminococcaceae* sp. [↑73.58%, FDR=1.79×10^-9^], among others. We used the nonparametric Mann-Whitney U test. The p-values were corrected for multiple testing through FDR.

**Table 3:** Bacteria with significant variability between normal and primary tumor tissues.

Species	FDR	Difference %	State (↑↓)
*Caldatribacterium* sp.	3.15 × 10^-11^	74.94%	↑
*Acidaminococcaceae* sp.	1.79 × 10^-9^	73.58%	↑
*Planctopirus* sp.	2.50 × 10^-9^	73.66%	↑
*Desulfobulbaceae* sp.	2.50 × 10^-9^	71.10%	↑
*Nocardia farcinica*	8.02 × 10^-9^	71.20%	↑

Noticeably, *Caldatribacterium* sp., *Acidaminococcaceae* sp. (amino acid cocci), and *Planctopirus* sp. are significantly more abundant in infiltrating tumor tissues as compared to normal tissues and are likely associated with the pathological development of hepatocellular carcinoma.

These results were substantiated by external molecular biology and clinical evidence from previous studies. For examples it was found that the increase in *Caldatribacterium* sp. leads to DNA damage in hepatic stellate cells, which then promotes the development of hepatocellular carcinoma through metabolites or toxins from intestinal bacteria (41). Studies also showed the presence of *Planctopirus* sp. bacterial infection in serum and tissues derived from patients with chronic liver disease – a common presage of hepatocellular carcinoma; the enrichment of *Planctopirus* sp. bacteria in the serum of patients with chronic liver disease is significantly higher than that of healthy individuals, suggesting that it may be associated with the development of chronic hepatitis B and primary liver cancer (42). The other bacteria identified in our study were also supported in the literature, where multiple species including *Gemmiger formicilis*, *Marithrix* sp., and *Haemophilus* sp. were found to be over-represented in in gut microbiome in patients with HCC (43).

We also found that the microbial diversity (Shannon Index) in the primary tumor tissue is significantly higher than that in normal tissue samples (see **Figure 5B**). This is somewhat contrary to the discoveries of higher gut microbiome diversity associated with healthy controls as known in many other related cancers (44).

### Joint Effects of Infiltrating Immune Cells and Bacteria On Hepatocellular Carcinoma

In order to further analyze the impact of tumor infiltrating immune cells and bacteria on the occurrence and development of liver cancer, we used the downloaded gene expression data and transcriptome data of 98 liver cancer samples, and calculated the corresponding bacterial abundance data by using the estimation method of microbial relative abundance in this paper. The PSO-SVR algorithm described in Section 3.1 was used to calculate the relative proportion of immune cells in liver cancer samples.

Then the logistic regression classification method was applied to three cases: case 1, only bacterial abundance data were used as the input feature; In case 2, only the immune cell proportion data were used as the input feature; and in case 3, both bacterial abundance and immune cell ratio were used as input characteristics. From the classification results of the three cases (see **Table 4**), the classification accuracy of using only a single input feature is lower - only bacteria: 0.70, only immune cell feature: 0.74, and both features: 0.84. No matter which data feature is added, the classification accuracy can be improved. After adding the immune cell proportion data, the classification accuracy is improved by 20% compared with case 1; After adding bacterial abundance data, the accuracy of classifier is improved by 13.51% compared with case 2. This shows that the bacteria and infiltrating immune cells in liver cancer tissue contain key characteristics for cancer diagnosis, which can be used as a reference index for clinical cancer diagnosis.

**Table 4:** Classification effect of liver cancer samples under different input features.

	Case1: Bacteria	Case2: Immune cell	Case3: Bacteria-Cell
**CIBERSORT**	0.68	0.64	0.80
	0.75	0.71	0.88
	0.67	0.67	0.88
**Average accuracy**	0.70	0.67	0.85
**PSO-SVR**	0.68	0.76	0.80
	0.75	0.79	0.75
	0.67	0.67	0.96
**Average accuracy**	0.70	0.74	0.84

## Conclusions

We performed a joint analysis of microbial and immune cell abundance in liver cancer tissue using a gene expression profile deconvolution algorithm combined with foreign read remapping. First, we filtered out reads from the RNA-Seq data that did not map to the human reference genome. Second, we assembled the single-ended unmapped reads with a reverse complementary sequence to the double-ended unmapped reads, and the processed reads were then mapped to the microbial reference library. Finally, the GRAMMy algorithm was introduced to calculate the relative abundance of microorganisms. This algorithm overcomes the problem of mapping one read to different microbial reference sequences owing to small reads and enables a more accurate calculation of relative abundance.

This complete procedure was then applied to RNA-seq samples of 98 patients with hepatocellular carcinoma, and we found that microorganisms including *Caldatribacterium* sp. and *Planctopirus* sp. differed significantly between normal and tumor tissues, which was also reported in the literature.

To study the degree of immune cell response to microorganisms and the effect on liver cancer in the human microenvironment, we introduced SVR and particle swarm algorithm to estimate the relative proportion of infiltrating immune cells based on the deconvolution model. The results of our study were validated by actual data and then compared with some immune cell counting algorithms, such as CIBESORTX, EPIC, and MCP-counter, and were found to be relatively more reliable.

The PSO-SVR algorithm was applied to the gene expression profile data of liver cancer samples. The differential analysis revealed significant differences in regulatory T cells, monocytes, and neutrophils between normal and tumor tissues. Finally, using the classification regression algorithm within machine learning, we found that adding microbial characteristics can improve the accuracy of liver cancer prediction.

This study has some limitations that result from the limited sample size. For example, a small number of samples affects statistical power, which we anticipate correcting in further studies. Also, liver cancer has different cancer subtypes, which are not subdivided in this study; The model does not take gene variation into account; otherwise, there can be new information to improve the accuracy.

## Data Availability Statement

Publicly available datasets were analyzed in this study. This data can be found here: https://portal.gdc.cancer.gov/repository and http://www.cancergenomicscloud.org/.

## Author Contributions

DA, LCX and YX conceived and designed the study. YX and GL performed the analyses and summarized the data. LCX and DA supervised the study. DA, YX and LCX wrote the manuscript with inputs from GL, XL, and YW. All authors have read and agreed to the published version of the manuscript.

## Funding

This work was supported by grants from the National Natural Science Foundation of China (61873027), open project of the National Engineering Laboratory for Agri-product Quality Traceability (No.AQT-2020-YB6), and GuangDong Basic and Applied Basic Research Foundation (2022A1515-011426).

## Conflict of Interest

The authors declare that the research was conducted in the absence of any commercial or financial relationships that could be construed as a potential conflict of interest.

## Publisher’s Note

All claims expressed in this article are solely those of the authors and do not necessarily represent those of their affiliated organizations, or those of the publisher, the editors and the reviewers. Any product that may be evaluated in this article, or claim that may be made by its manufacturer, is not guaranteed or endorsed by the publisher.
