# Supplementary material for: Joint Analysis of Microbial and Immune Cell Abundance in Liver Cancer Tissue Using a Gene Expression Profile Deconvolution Algorithm Combined With Foreign Read Remapping
Source: Front Immunol. 2022 Apr 14;13:853213. doi: 10.3389/fimmu.2022.853213 (PMC9047545; doi:10.3389/fimmu.2022.853213)
Supplement: Supplementary file 1 [file Table_1.docx]

Supplementary Material

# Supplementary Figures and Tables

## Supplementary Tables

**Supplementary Table 1.** **Specific information on the three sets of validation data**

|  | **Sample size** | **Data set** | **Quantification methods** |
| --- | --- | --- | --- |
| PBMC-FC | 20 | CIBERSORT | Flow cytometry |
| CRC-IC | 10 | GSE64385 | Immunohistochemistry |
| Melanoma-scRNA | 19 | CIBERSORTX | Single cell sequencing |

1. PBMC-FC: Gene expression profiles of blood samples from 20 adult subjects, data from the article CIBERSORT, and the relative proportions of 4 kinds of immune cells determined through flow cytometry by Newman et al.
2. CRC-IC: Gene expression profiles of 12 patients with colorectal cancer collected from the National Center for Biotechnology Information Search database (NCBI database) under the GEO Datasets GSE64385. GEO Datasets GSE64385 contains the relative proportions of 6 kinds of immune cells in 12 samples determined through immunohistochemistry.
3. Melanoma-scRNA: Gene expression profiles of 19 melanoma samples and the proportion of immune cells obtained through single-cell sequencing and data obtained from CIBERSORTX. The relative proportion of immune cells was determined through single-cell sequencing by Newman et al.

**Supplementary Table 2. PBMC-FC: Algorithm Comparison**

|  | **PBMC-FC** | | |
| --- | --- | --- | --- |
|  | **Root mean square error** | **Pearson** | **Spearman** |
| CIBERSORTX | 0.0784 | 0.6302 | 0.6743 |
| EPIC | 0.0821 | 0.6292 | 0.5864 |
| MCP-counter | 0.0884 | 0.5643 | 0.5209 |
| PSO-SVR | 0.0736 | 0.8851 | 0.8875 |

**Supplementary Table 3. CRC-IC: Algorithm Comparison**

|  | **CRC-IC** | | |
| --- | --- | --- | --- |
|  | **Root mean square error** | **Pearson** | **Spearman** |
| CIBERSORTX | 0.0823 | 0.6423 | 0.7021 |
| EPIC | 0.0913 | 0.6312 | 0.6742 |
| MCP-counter | 0.1021 | 0.5603 | 0.5643 |
| PSO-SVR | 0.0724 | 0.6742 | 0.5788 |

**Supplementary Table 4. Melanoma-scRNA: Algorithm Comparison**

|  | **Melanoma-scRNA** | | |
| --- | --- | --- | --- |
|  | **Root mean square error** | **Pearson** | **Spearman** |
| CIBERSORTX | 0.0976 | 0.6992 | 0.6523 |
| EPIC | 0.1123 | 0.6482 | 0.6623 |
| MCP-counter | 0.1235 | 0.5467 | 0.5235 |
| PSO-SVR | 0.0329 | 0.9799 | 0.9236 |

**Supplementary Table 5. Immune cells with significant differences in tumor and normal solid tissue samples**

| **Cell type** | **P-value** | **Q-value (adjusted FDR)** | **(tumor-normal)/tumor** |
| --- | --- | --- | --- |
| T cells regulatory (Tregs) | 7.53E-09 | 1.58E-07 | 59.87% |
| Mast cells resting | 9.68E-08 | 6.77E-07 | 67.90% |
| Monocytes | 8.77E-08 | 6.77E-07 | -150.07% |
| Macrophages M2 | 3.47E-07 | 1.51E-06 | -47.82% |
| Neutrophils | 4.57E-06 | 1.37E-05 | -363.69% |
| Plasma cells | 3.98E-06 | 1.37E-05 | -142.38% |
| T cells gamma delta | 4.79E-05 | 0.000125708 | -378.59% |
| Dendritic cells resting | 0.000176196 | 0.000411124 | 99.36% |
| Macrophages M0 | 0.000518513 | 0.001088876 | 44.39% |
| Macrophages M1 | 0.010984876 | 0.020971127 | -27.29% |
| Dendritic cells activated | 0.027831419 | 0.048704983 | 91.69% |
| NK cells activated | 0.100417384 | 0.162212697 | 22.14% |
| B cells memory | 0.162251439 | 0.243377159 | 89.22% |
| B cells naive | 0.229658052 | 0.321521273 | 16.17% |
| Eosinophils | 0.251894834 | 0.33061197 | -352.44% |
| NK cells resting | 0.287629982 | 0.355307624 | 39.46% |
| T cells CD4 memory activated | 0.305647402 | 0.356588636 | 59.42% |
| T cells CD4 memory resting | 0.397736293 | 0.439603271 | 8.91% |
| T cells CD8 | 0.777229536 | 0.816091012 | 11.30% |
| T cells follicular helper | 0.827419531 | 0.827419531 | 34.44% |

# Supplementary Algorithm Formula

Particle swarm optimization algorithm mainly has two vectors -$v_{i}$and $x_{i}$, and its iterative update formula is:

$$v_{i+1}=qv_{i}+c_{1}r_{1}\left( p_{\mathrm{ibest}}-x_{i} \right)+c_{2}r_{2}\left( g_{\mathrm{best}}-x_{i} \right)$$

$$x_{i+1}=x_{i}+v_{i+1}$$

Where $v_{i}$and $x_{i}$ represent the velocity vector and position vector of the ith particle respectively, each gene is regarded as a particle respectively, and n represents the size of the population, specifically the number of genes; q is a non-negative inertia factor. The larger its value is, the stronger the global optimization ability is and the weaker the local optimization ability is; $c_{1}$ and $c_{2}$are learning factors, general $\mathbf{c}_{\mathbf{1}}\mathbf{=}\mathbf{c}_{\mathbf{2}}\mathbf{=2}$; $\mathbf{r}_{\mathbf{1}}$ and $\mathbf{r}_{\mathbf{2}}$ both represent random coefficients belonging to [0,1]; $p_{\mathrm{ibest}}$ represents the individual optimal value of the ith particle, and $g_{\mathrm{best}}$ is the global optimal value. We provide more detailed parameter explanation and algorithm flow in the supplementary materials.

In order to speed up the iteration, the Q value should decrease with the increase of the number of iterations. In this embodiment, it is defined as:

$$q=q_{\min}+\left( \mathrm{iter}_{\max}-\mathrm{iter}_{\min} \right)*\frac{q_{\max}-q_{\min}}{\mathrm{iter}_{\max}}$$

Where $q_{\min}$ and $q_{\max}$ represent the upper and lower limits of q respectively, $\mathrm{iter}_{\max}$ represents the maximum number of iterations, and $\mathrm{iter}_{\min}$ represents the current number of iterations.

The gene expression profile data is substituted into the above iterative process and fitted to a new regression function as below; $a_{i}$ and $\dot{a}$ are Lagrange multipliers.

$$f\left( x \right)=w^{*}v\left( x \right)+b^{*}=\sum_{i=1}^{n} \left( \dot{a}-a_{i} \right)\varphi\left( x_{i} \right)\varphi\left( x \right)+b^{*}$$

$$=\sum_{i=1}^{n} \left( \dot{a}-a_{i} \right)k\left( x_{i},x \right)+b^{*}$$

For the solution of this problem, considering that the measurement standard of the accuracy of calculating the relative proportion of immune cells is the size of the loss function, in order to minimize the loss function, the following algorithm principle is introduced.

Through a nonlinear transformation $\varphi(x)$, X is mapped to the high-dimensional space, which is the ith element of the n-dimensional space. In the high-dimensional space, the theory has a linear function $f(x)$, which can well describe the relationship between the input data and the output data. Such a linear function becomes the SVR function:

$$\begin{aligned} f\left( x \right)=w^{T}\varphi\left( x \right)+b,\varphi：R^{n}\to F,w\in F \end{aligned}$$

The following structural risk functions are defined according to SVR regression error:

$$R_{\mathrm{reg}}=\frac{1}{2}\left\| w \right\|^{2}+C\frac{1}{n}\sum_{i=1}^{n} \left| y-f\left( x \right) \right|_{\varepsilon}$$

$$\left| y-f\left( x \right) \right|_{\varepsilon}=\left\{ \begin{aligned} 0, \left| y-f\left( x \right) \right|\leq\varepsilon\\ \left| y-f\left( x \right) \right|<\varepsilon,\mathrm{others} \end{aligned} \right.$$

Furthermore, the original problem is equivalent to the minimization cost functional：

$$\begin{aligned} \min\frac{1}{2}\left\| w \right\|^{2}+C\sum_{i=1}^{n} {(§}_{i}+§_{i}^{*}) \end{aligned}$$

s.t.

$y_{i}-w^{T}\varphi\left( x \right)-b\leq\varepsilon+§_{i}^{*}$,i=1,2,…,n

$-y_{i}-w^{T}\varphi\left( x \right)+b\leq\varepsilon+§_{i}$,i=1,2,…n

$$§_{i}^{*},§_{i}\geq0,i=1,2,\ldots,n$$

$§_{i} \mathrm{and} §_{i}^{*}$ represents two relaxation variables, whose purpose is to make the solution of the above formula exist. When solving the optimization problem, it is first transformed into solving the dual problem, and then the Lagrange function is introduced. By calculating the partial derivatives of Lagrange multipliers $a_{i}$,$a_{i}^{*}$, w and b, each partial derivative is equal to zero, Finally, the KKT (karush-Kuhn-Tucker conditions) condition is used to solve the support vector regression (SVR) model for deconvolution to solve the proportion of immune cells (also known as the proportion counting model of immune cells):

$$\begin{aligned} f\left( x \right)=\sum_{i=1}^{n} \left( a_{i}-a_{i}^{*} \right)k\left( x_{i},x \right)+b \end{aligned}$$

$$k\left( x_{i},x \right)=\varphi(x_{i})\varphi(x)$$

$k\left( x_{i},x \right)$ is the kernel function satisfying Mercer condition, in which Mercer condition refers to a condition as the kernel function, and any positive semidefinite symmetric function can be used as the kernel function. The solution of the final model consists of w and b.

$$\begin{aligned} w=\sum_{i=1}^{n} \left( a_{i}-a_{i}^{*} \right)\varphi\left( x \right) \end{aligned}$$

$$\begin{aligned} b=y_{i}+\varepsilon-\sum_{i=1}^{n} \left( a_{i}-a_{i}^{*} \right)k\left( x_{i},x \right) \end{aligned}$$
